# Effects of Paternal Obesity on Fetal Development and Pregnancy Complications: A Prospective Clinical Cohort Study

**DOI:** 10.3389/fendo.2022.826665

**Published:** 2022-03-14

**Authors:** Jing Lin, Wei Gu, Hefeng Huang

**Affiliations:** ^1^ International Peace Maternity and Child Health Hospital, School of Medicine, Shanghai Jiao Tong University, Shanghai, China; ^2^ Shanghai Key Laboratory of Embryo Original Diseases, Shanghai, China; ^3^ Obstetrics and Gynecology Hospital, Fudan University, Shanghai, China

**Keywords:** paternal obesity, macrosomia, SGA, preeclampsia, cohort study

## Abstract

**Objectives:**

To evaluate the association between paternal obesity and fetal development and pregnancy complications.

**Study Design:**

This prospective cohort clinical trial analyzed data from 7683 women with singleton pregnancies. All study subjects were sequentially divided into four groups based on paternal BMI. We compared the differences in fetal growth and pregnancy complications between different paternal BMI groups by univariate logistic regression and independent t-test. Finally, the independent predictors of SGA and macrosomia were determined.

**Results:**

The incidences of preeclampsia, cesarean section, SGA, macrosomia, and postpartum hemorrhage in the paternal obesity group were significantly higher than the normal BMI group. With the increase of paternal BMI, fetal ultrasound measurement parameter, neonatal and placental weight showed an increasing trend (trend P < 0.05). However, these differences disappeared in the obese group. The test for interaction showed the effect of paternal obesity on SGA and macrosomia was significantly affected by maternal obesity. We also found paternal obesity was an independent predictor of both SGA and macrosomia. Based on the above results, we plotted the Nomograms for clinical prediction.

**Conclusion:**

Paternal obesity can affect fetal growth parameters and placental development, which has an adverse impact on pregnancy outcomes. Optimizing the paternal BMI will help improve the health of the next generation.

## Introduction

Over the past 40 years, a global debate on population obesity has emerged, and many countries are now suffering from an obesity epidemic. Worldwide, more than 155 million people are overweight, and about 40 million children and adolescents are obese (1). The effects of obesity on individuals are well documented and involve an increased risk of cardiovascular disease, diabetes, and stroke. In recent decades, the rapid increase in obesity cannot be explained exclusively by genomic DNA mutations or selection (2). This suggests the involvement of other causes, including gene expression of epigenetic modifications, which can occur over the life cycle of numerous individuals in a population and can therefore be immediately transmitted to a large number of offspring in the next generation (3). Although the developmental planning of mothers for the health of their offspring is widely accepted, women are thus described as being primarily responsible for the intergenerational transmission of obesity (4, 5). However, through a structural review of recent studies examining epigenetic and social mechanisms of obesity risk transmission, we suggest that the role of fathers in influencing obesity risk in early childhood has been underestimated. Previous reports did not consider the potential impact of paternal obesity, which would cause bias (6, 7). Although the design and execution of epidemiological studies on patrilineal exposure before pregnancy face challenges, especially in terms of selection bias and recruitment, we believe it is feasible and necessary (8). There is a growing awareness that parental obesity may have a negative impact on fetal development (9, 10). A review of studies on mouse models and human male obesity has clarified that male obesity has a significant negative impact on sperm motility, DNA damage and embryos in various key early developmental stages (11). Paternal obesity can not only induce epigenetic changes of sperm before conception, but also affect the development process during pregnancy (12). Recent data also suggest that paternal nutrition and weight status can alter placental function in offspring. In a high-fat diet-induced mouse model of obesity, paternal obesity is associated with placental weight loss, fetal growth restriction, and changes in placental genes expression associated with lipid metabolism. Although few studies have assessed the effects of paternal obesity on human placental function, several research groups have reported showing that paternal body mass index (BMI) is a predictor of offspring placental and birth weight (13). Future human cohort studies must collect reliable health information for both partners and include it appropriately in statistical analyses to determine true parental impact. At present, no study has used a large birth cohort to evaluate the impact of paternal obesity on fetal development and perinatal complications. Therefore, the aim of this study was to investigate the influence of paternal obesity on fetal development (BPD, FL, APAD, TAD, AC, birthweight, and body length), placenta development (weight and area), pregnancy complications (GDM, ICP, preeclampsia, preterm delivery), and pregnancy outcomes (delivery mode, neonatal asphyxia, postpartum hemorrhage).

## Material and Methods

### Participants

We invited a total of 8210 Chinese women who delivered their babies at the obstetric department of the International Peace Maternity and Child Health Hospital between November 2020 to August 2021 to participate in the study. Pregnant women with singleton pregnancies who had regular antenatal examinations were included in this prospective study. In the present study, only single pregnancy outcomes were investigated. After excluding cases that did not meet the inclusion criteria or were unwilling to follow-up, we finally included 7767 remaining cases. The Ethics Committee of the International Peace Maternity and Child Health Hospital approved the study procedures (reference number GKLW 2021-23). This study was registered with the China Clinical Trials Registry (www.clinicaltrials.gov) (registration number ChiCTR2000037885). All participants provided written informed consent and the ethics committee approved the consent procedure.

### Inclusion and Exclusion Criteria

The inclusion criteria for this prospective study were:(1) Chinese nationality, (2) singleton pregnancy, (3) maternity files had been established in hospital, (4) regular antenatal examination, (5) complete follow-up data are available, (6) signed informed consent forms.

The exclusion criteria were:(1) foreign nationality, (2) multiple pregnancies, (3) inability to have regular maternity visits, (4) other hospital deliveries, (5) with contraindications to pregnancy such as gynecological oncology.

### BMI Grouping

In 2006, the China Obesity working group, China office, international society for Life Sciences summarized and analyzed the relationship between body mass index and the prevalence of related diseases according to the large-scale measurement data of the Chinese population, put forward the limits for Chinese adults to judge the degree of overweight and obesity. This study adopted the body mass index (BMI) in the guidelines for the prevention and control of overweight and obesity in Chinese adults. The cut-off point value was used to determine the degree of obesity. The accuracy of height is 0.1cm and the accuracy of weight is 0.1kg. BMI = weight (kg)/height^2^ (m^2^). According to the suggestions, BMI is divided into four groups (14, 15): BMI < 18.5 is underweight, 18.5 ≤ BM < 24 is normal, 24 ≤ BMI < 28 is overweight, and BMI ≥ 28 is obesity.

### Clinic Data Collection

All the participants’ data were recorded by assigned persons and filed electronically, including age, gravidity and parity, BMI, personal and family history, IVF-ET, multiple pregnancies, weight gain during pregnancy, gestational age at delivery, pregnancy complications. All participants received regular follow-up visits. Weight gain during pregnancy was determined as the difference between the pre-pregnancy weight and the last measured weight before delivery. Paternal BMI figures were calculated from self-reported height and weight. Biometric data for newborns and placentas are routinely measured immediately after delivery. The birth weight, body length, placental weight and placental area of newborns were collected and recorded. Delivery mode and pregnancy outcomes were also recorded after the delivery, including postpartum hemorrhage, mode of delivery, Apgar score and amniotic fluid volume. We also took some measures to minimize observer bias. For example, we used masking to hide the research purpose of all observations and used different data collection sources for data triangulation. We used multiple observers and conducted unified training to ensure the reliability of data records and standardized observation procedures.

### Ultrasound Measurements During Pregnancy

Collect and record the fetal biparietal diameter, femoral length, transverse abdominal diameter, anterior-posterior abdominal diameter and abdominal circumference measured by ultrasound (16) within one week before delivery. Professional ultrasound doctors conduct ultrasonic diagnostic examination and analysis to ensure the quality of data. The diameter of biparietal bone is measured from the outer edge of parietal bone near the probe to the inner edge of parietal bone on the other side on the cross section of fetal brain. The main signs include hyaline membrane cavity, thalamus, third ventricle and accumbent compartment. Using the ellipse function of the ultrasonic instrument, measure the head circumference on the same plane as the biparietal diameter. The abdominal circumference was measured in a plane perpendicular to the level of the fetal umbilical plexus, including the spine, gastric vesicles, liver, umbilical vein, skin, and subcutaneous fat. In the scan, the femoral length was measured from the greater trochanter to the lateral condyle, and the bones at both ends were clearly visible.

### Definition of Pregnancy Complications

Pre-eclampsia is defined according to the International Society for the Study of Hypertension in Pregnancy (17). Previously normotensive women should have a systemic systolic blood pressure ≥140 mmHg and/or a diastolic blood pressure ≥90 mmHg after 20 weeks of gestation, both at least 4 hours apart. Hypertension should be accompanied by ≥300 mg of proteinuria in 24 hours or, if no 24-hour urine collection is available, 2 readings of at least ++ on a midstream or catheter urine specimen. Gestational age is calculated from the first day of the last normal menstrual period and is confirmed by an ultrasound scan in the first trimester of pregnancy. SGA refers to the birth weight is under the lightest 10% of birth weights for all babies born at the same number of gestation weeks of pregnancy. Macrosomia is defined as the birth weight of a newborn equal to or greater than 4000 grams. Preterm birth is defined as birth before 37 weeks of pregnancy. Postpartum Hemorrhage is defined as blood loss ≥1000 ml after vaginal or cesarean delivery (18, 19). GDM is defined as any degree of glucose intolerance with onset or first recognition during pregnancy. ICP is characterized by pruritus and elevated serum bile acid concentrations and usually develops in the third trimester of pregnancy and resolves rapidly after delivery (20).

### Statistical Analysis

Data were analyzed with SPSS version 21.0 (IBM Corp., Armonk, NY) and R software version 3.6.1 (R Development Core Team, July 2019; http://www.r-project.org). Quantitative data were expressed as means ± standard deviation and compared using the independent samples t-test. Qualitative data were expressed as rates and compared using the chi-square test. Univariate logistic regression was used to compare pregnancy complications and outcomes in different paternal BMI groups by using forward stepwise. Multivariate regression analysis was used to adjust for confounding variables known to independently affect SGA and macrosomia. Risk ratios (ORs) with 95% confidence intervals (CIs) were calculated to identify risk factors and assess their impact. The nomogram was established based on results of the multivariate logistic regression analysis using software R 3.0.3 by using the “rms” package. P-value < 0.05 was considered significant.

## Result

### Description of the Cohort

**Figure 1:**
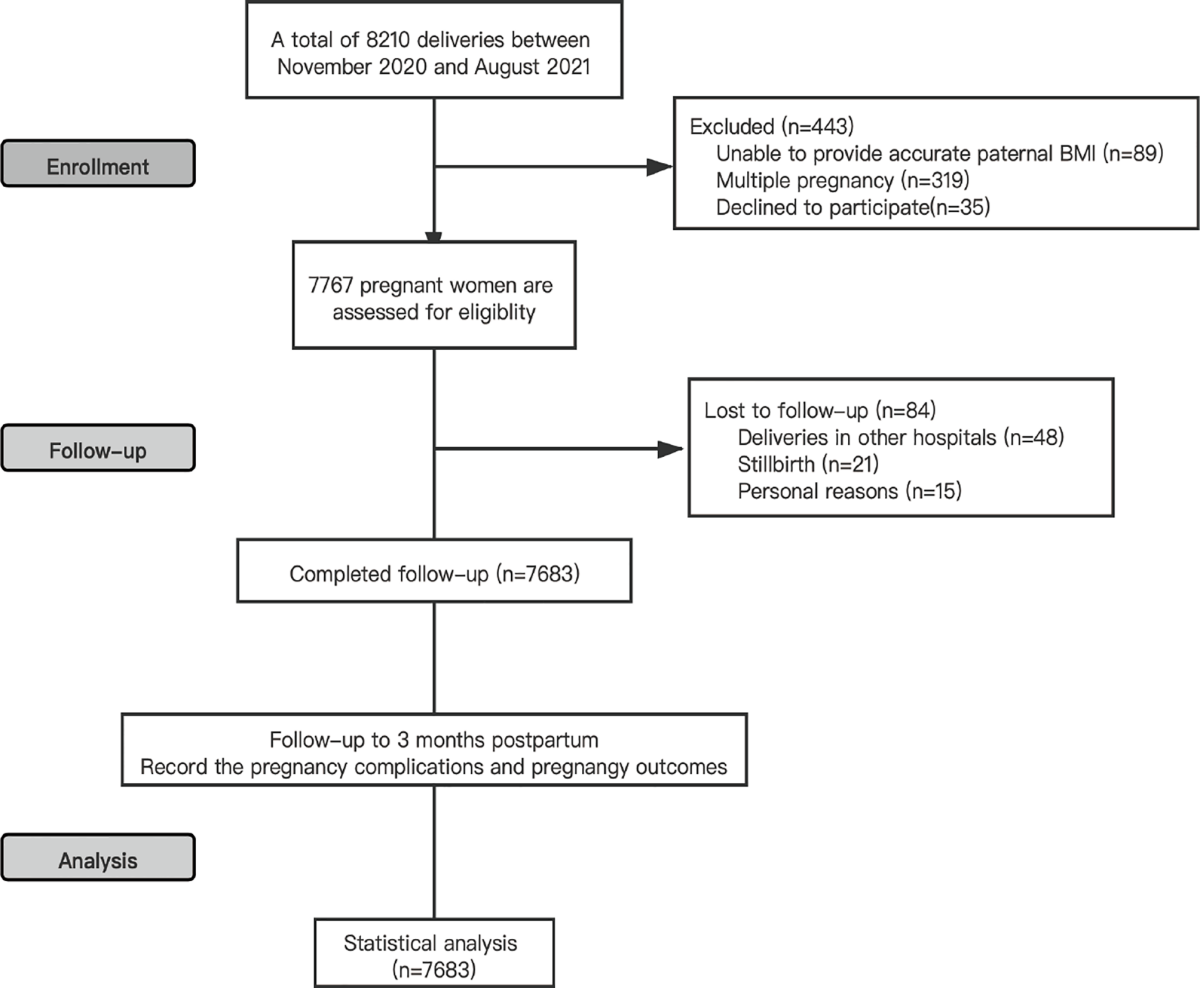
Flowchart of participants in the study.

Of the 7767 eligible women, 84 lost the chance of follow-up (48 delivered in other hospitals, 21 stillbirths and 15 withdrew from follow-up for personal reasons). **Figure 1** showed the progress of participants through the study. Finally, 7683 participants with a singleton pregnancy completed the whole follow-up process. We finally included 7683 father/mother/child triplets with 3932 newborn boys and 3751 newborn girls, respectively. The mean paternal age was 32.7 ± 4.6 years and their mean body mass index was 24.1kg/m^2^, whereas the mean maternal age was 31.3 ± 3.8 years and the mean BMI at early pregnancy was 21.1 kg/m^2^. At the pregravid assessment, paternal BMl increased progressively across the groups from the lowest to the highest of BMI. Among them, a total of 201 (2.7%) were in the underweight group, 3813 (49.6%) were in the normal BMI group, 2913 (37.9%) were in the overweight group and 756 (9.8%) were in the obesity group. Maternal BMI was also divided into four groups, including 1116 in underweight (14.5%), 5495 in normal (71.5%), 893 in overweight (11.6%) and 179 in obesity (2.3%). The baseline characteristics of the study population (such as parental age, gravidity, parity, parental education, mode of conception, maternal BMI and maternal disease history) are shown in **Table 1**, stratified into four groups based on paternal BMI.

**Table 1:** Baseline characteristics of patients in the study.

		Paternal BMI (kg/m^2^)			
		Underweight (n = 201)	Normal (n = 3813)	Overweight (n = 2913)	Obesity (n = 756)
**Maternal age (y)**		29.79 ± 3.90	31.17 ± 3.68	31.50 ± 3.77	31.56 ± 3.87
**Paternal age (y)**		30.97 ± 4.03	32.54 ± 4.62	32.97 ± 4.56	33.08 ± 4.86
**Gravidity (%)**	<3	172 (85.6%)	3066 (80.4%)	2300 (79.0%)	595 (78.7%)
	≥3	29 (14.4%)	747 (19.6%)	613 (21.0%)	161 (21.3%)
**Parity (%)**	<2	171 (85.1%)	2793 (73.2%)	2085 (71.6%)	546 (72.2%)
	≥2	30 (14.9%)	1020 (26.8%)	828 (28.4%)	210 (27.8%)
**Maternal education (%)**	High school and below	22 (10.9%)	236 (6.2%)	167 (5.7%)	68 (9.0%)
	Bachelor’s degree	143 (71.2%)	2601 (68.2%)	1984 (68.1%)	532 (70.4%)
	Master degree or above	36 (17.9%)	976 (25.6%)	762 (26.2%)	156 (20.6%)
**Paternal education (%)**	High school and below	15 (7.4%)	225 (5.9%)	160 (5.5%)	60 (7.9%)
	Bachelor’s degree	158 (78.6%)	2501 (65.6%)	1992 (68.4%)	521 (68.9%)
	Master degree or above	28 (14.0%)	1087 (28.5%)	761 (26.1%)	175 (23.2%)
**Conception (%)**	Natural	184 (91.5%)	3451 (90.5%)	2607 (89.5%)	660 (87.3%)
	IVF	17 (8.5%)	361 (9.5%)	306 (10.5%)	96 (12.7%)
**Maternal BMI** **(kg/m^2^)**	Underweight	55 (27.4%)	603 (15.8%)	373 (12.8%)	85 (11.2%)
	Normal	131 (65.2%)	2783 (73.0%)	2077 (71.3%)	504 (66.7%)
	Overweight	11 (5.5%)	374 (9.8%)	382 (13.1%)	126 (16.7%)
	Obesity	4 (1.9%)	53 (1.4%)	81 (2.8%)	41 (5.4%)
**Maternal medical history (%)**	Chronic hypertension	0 (0%)	17 (0.4%)	15 (0.5%)	7 (0.9%)
	Diabetes	0 (0%)	0 (0%)	3 (0.1%)	2 (0.3%)
	Heart disease	0 (0%)	2 (0.1%)	2 (0.1%)	2 (0.3%)

Data are given as number (percentage) or as the mean ± SD. BMl, body mass index.

### Effects of Paternal BMI on Pregnancy Complications and Delivery Outcomes

**Table 2** showed the results of univariate logistic regression, which compared the differences of pregnancy complications and delivery outcomes in different paternal BMI groups by forward stepwise. The intergroup p value represented the univariate logistic regression results compared with the reference (normal BMI group). The results showed that the incidences of preeclampsia, SGA, macrosomia, cesarean and postpartum hemorrhage in paternal obesity group were significantly higher than in normal BMI group. The rates of macrosomia and cesarean section in paternal overweight group were also significantly higher than those in normal BMI group. And the SGA rate in paternal underweight group was significantly higher than in normal BMI group. In addition, trend p values represented the linear relationship between paternal BMI and pregnancy outcomes across the different categories. It reflected that with the increase of paternal BMI, the higher the rate of preeclampsia, SGA, macrosomia, and cesarean section.

**Table 2:** Univariate logistic regression comparing pregnancy outcomes in different paternal BMI groups by using forward stepwise.

		Underweight (BMI <18.5)	Normal (BMI = 18.5~23.9)	Overweight (BMI = 24.0~27.9)	Obesity (BMI ≥ 28)	P value for trend^b^
**GDM**	Incidence (%)	17.4	14.3	15.0	16.5	0.821
	P^a^	0.221	Reference	0.438	0.112	
	OR (95%CI)	1.264 (0.868-1.841)	1	1.055 (0.921-1.210)	1.188 (0.961-1.469)	
**ICP**	Incidence (%)	1.0	0.7	0.8	0.5	0.851
	P^a^	0.569	Reference	0.627	0.690	
	OR (95%CI)	1.523 (0.358-6.474)	1	1.153 (0.649-2.049)	0.806 (0.280-2.323)	
**Preeclampsia**	Incidence (%)	3.0	2.2	2.8	4.6	0.001^*^
	P^a^	0.484	Reference	0.107	<0.001^*^	
	OR (95%CI)	1.350 (0.582-3.127)	1	1.286 (0.947-1.747)	2.129 (1.425-3.181)	
**Premature birth**	Incidence (%)	4.0	3.9	4.1	3.2	0.745
	P^a^	0.925	Reference	0.603	0.415	
	OR (95%CI)	1.036 (0.501-2.141)	1	1.068 (0.833-1.369)	0.833 (0.537-1.293)	
**SGA**	Incidence (%)	18.9	2.9	3	8.5	<0.001^*^
	P^a^	<0.001*	–	0.806	<0.001*	
	OR (95%CI)	7.848 (5.257-11.717)	Reference	0.806 (1.036-0.779)	3.113 (2.264-4.282)	
**Macrosomia**	Incidence (%)	2.5	3.1	5.7	6.6	<0.001^*^
	P^a^	0.601	–	<0.001^*^	<0.001^*^	
	OR (95%CI)	0.785 (0.317-1.943)	Reference	1.872 (1.473-2.379)	2.180 (1.552-3.061)	
**Cesarean delivery**	Incidence (%)	39.8	41.8	46.2	47.8	<0.001^*^
	P^a^	0.580	Reference	<0.001^*^	0.002^*^	
	OR (95%CI)	0.921 (0.690-1.231)	1	1.199 (1.088-1.321)	1.274 (1.089-1.489)	
**Neonatal asphyxia**	Incidence (%)	0.5	0.6	0.4	0.4	0.433
	P^a^	0.884	Reference	0.462	0.542	
	OR (95%CI)	0.862 (0.116-6.424)	1	0.772 (0.388-1.536)	0.687 (0.205-2.300)	
**Postpartum hemorrhage**	Incidence (%)	0.3	0.5	0.3	0.8	0.178
	P^a^	0.601	Reference	0.879	0.046^*^	
	OR (95%CI)	1.728 (0.222-13.452)	1	1.071 (0.443-2.588)	2.765 (1.019-7.500)	

BMl, body mass index; GDM, gestional diabetes mellitude; ICP, intrahepatic cholestasis of pregnancy; SGA, small for gestational age; OR, odds ratio;95% CI, 95% confidence interval of the estimated trend.

a The p-values taken from univariate logistic regression compared with the referent (normal BMI).

b P values for trend across categories of different paternal BMI.

*P<0.05 was considered statistically significant.

### Effect of Paternal BMI on Fetal Growth and Placental Development

Ultrasonography was performed within one week before delivery. **Table 3** illustrates the comparison of fetal ultrasound parameters and postpartum measurements in different paternal BMI groups. The trend test results showed that with the increase of paternal BMI, fetal BPD (biparietal diameter), FL (femoral length), TAD (transverse abdominal diameter), APAD (anteriorposterior abdominal diameter), AC (abdominal circumference), birth weight, placental weight and placental area showed an increasing trend (trend P < 0.05). Compared with the normal paternal BMI group, BPD, AC, placental weight and placental area decreased in the underweight group, and increased significantly in the overweight group. However, the above indicators showed no significant difference in the obesity group compared with the control group.

**Table 3:** Comparison of fetal growth and placental development in different paternal BMI groups.

Measurements		Paternal BMI (kg/m^2^)					P for trend^b^
		Group	Underweight (BMI < 18.5)	Normal (BMI = 18.5~23.9)	Overweight (BMI = 24.0~27.9)	Obesity (BMI ≥ 28)	
**Ultrasound measurements**	BPD	Mean ± SD	93.04 ± 5.12	94.20 ± 3.73	94.59 ± 3.79	94.51 ± 3.62	<0.001^*^
		P^a^	0.019^*^	[reference]	0.002^*^	0.106	
	FL	Mean ± SD	68.73 ± 4.05	69.45 ± 2.89	69.61 ± 2.99	69.72 ± 2.83	0.001^*^
		P^a^	0.011^*^	[reference]	0.108	0.07	
	APAD	Mean ± SD	101.50 ± 7.50	103.81 ± 6.81	104.28 ± 6.97	104.14 ± 7.08	<0.001^*^
		P^a^	0.001^*^	[reference]	0.038^*^	0.362	
	TAD	Mean ± SD	99.68 ± 7.28	101.42 ± 6.60	101.71 ± 6.77	102.04 ± 6.34	0.001^*^
		P^a^	0.007^*^	[reference]	0.191	0.069	
	AC	Mean ± SD	316.86 ± 20.60	323.24 ± 17.70	324.44 ± 18.47	324.73 ± 18.21	<0.001^*^
		P^a^	0.002^*^	[reference]	0.044^*^	0.115	
**Placental measurements**	Placental weight	Mean ± SD	564.68 ± 25.66	607.78 ± 34.33	616.42 ± 42.63	613.59 ± 36.17	<0.001^*^
		P^a^	<0.001^*^	[reference]	<0.001^*^	0.125	
	Placental area	Mean ± SD	231.16 ± 15.87	241.76 ± 14.09	245.02 ± 16.12	242.76 ± 16.14	0.015^*^
		P^a^	0.025^*^	[reference]	0.045^*^	0.692	
	Umbilical cord length	Mean ± SD	58.24 ± 7.27	59.88 ± 8.85	57.94 ± 6.28	63.61 ± 9.79	0.428
		P^a^	0.793	[reference]	0.236	0.332	
**Newborn measurements**	Birthweight	Mean ± SD	3116.22 ± 441.712	3284.65 ± 407.59	3331.12 ± 422.06	3339.23 ± 429.72	<0.001^*^
		P^a^	<0.001^*^	[reference]	<0.001^*^	<0.001^*^	
	Body length	Mean ± SD	49.29 ± 1.64	49.74 ± 1.48	49.83 ± 1.47	49.88 ± 1.51	0.747
		P^a^	<0.001^*^	[reference]	0.008^*^	0.014^*^	

BMl, body mass index; BPD, biparietal diameter; FL, femoral length; TAD, transverse abdominal diameter; APAD, anteriorposterior abdominal diameter; AC, abdominal circumference

The data are presented as the mean values ± standard deviations.

a The p-values taken from independent samples t-test and the reference group in this analysis was normal BMI group.

b P values for trend across categories of different paternal BMI.

*P < 0.05 was considered statistically significant.

### Value of Paternal Obesity as an Independent Factor in Predicting Macrosomia and SGA

**Figure 2:**
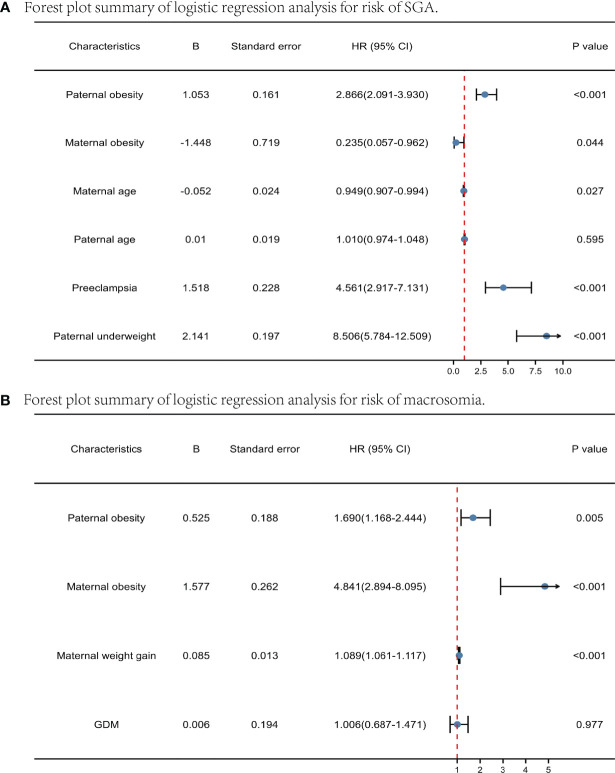
Forest plot summary of logistic regression analysis for risk of SGA and macrosomia. Predictors of SGA **(A)** and macrosomia **(B)** pregravid and before pregnancy. Data were presented as odds ratio per standard deviation change in the indicated variable.

Firstly, paternal BMI and the risk of SGA/macrosomia was analyzed by univariate and multivariate logistic regression, stratified by maternal BMI (**Table 4**). The test for interaction showed that the effect of paternal obesity on SGA and macrosomia was significantly affected by maternal obesity (P < 0.005). For maternal underweight, paternal underweight can significantly increase the incidence of SGA. For maternal BMI normal and overweight, paternal underweight and obesity can significantly increase the occurrence of SGA, while paternal overweight and obesity can significantly increase the incidence of macrosomia. However, for maternal obesity, paternal BMI had little effect on the incidence of both SGA and macrosomia. We next performed logistic regression analysis to determine the independent determinants of macrosomia and SGA. **Figure 2** represented the forest plot of logistic regression analyses of the risk of SGA and macrosomia. (A) and (B) represented independent predictors of SGA and macrosomia respectively. Data were presented as odds ratio per standard deviation change in the indicated variable. For SGA, the important independent predictors were paternal obesity (P<0.001), maternal obesity (P=0.044), preeclampsia (P<0.001) and paternal underweight (P<0.001). For macrosomia, the independent predictors were paternal obesity (P=0.005), maternal obesity (P<0.001) and maternal weight gain during pregnancy (P<0.001). The result showed that both paternal obesity and maternal obesity were important independent predictors in macrosomia and SGA.

**Table 4:** Paternal BMI and the risk of SGA/macrosomia was analyzed by logistic regression, stratified by maternal BMI.

Maternal BMI		Paternal BMI		Crude		Adjusted		P for Interaction^c^
		Group	Incidence (%)	OR (95% CI)^a^	P value	OR(95% CI)^b^	P value	
**SGA**	**Underweight**	Underweight	5.0	9.324 (4.766-18.238)	<0.001*	9.435 (4.733-18.809)	<0.001*	<0.001
		Normal	53.7	Ref		Ref		
		Overweight	33.6	0.715 (0.382-1.338)	0.294	0.708 (0.373-1.342)	0.290	
		Obesity	7.7	0.834 (0.834-0.287)	0.738	0.645 (0.211-1.975)	0.443	
	**Normal**	Underweight	2.4	5.416 (2.931-10.007)	<0.001*	4.962 (2.664-9.241)	<0.001*	
		Normal	50.4	Ref		Ref		
		Overweight	37.9	1.113 (0.760-1.626)	0.584	1.128 (0.770-1.654)	0.537	
		Obesity	9.3	3.781 (2.493-5.734)	<0.001*	3.837 (2.517-5.850)	<0.001*	
	**Overweight**	Underweight	1.3	23.357 (4.834-112.855)	<0.001*	19.147 (3.518-104.211)	0.001*	
		Normal	41.7	Ref		Ref		
		Overweight	42.9	1.637 (0.588-4.555)	0.346	1.718 (0.610-4.837)	0.306	
		Obesity	14.1	8.990 (3.424-23.602)	<0.001*	8.861 (3.285-23.899)	<0.001*	
	**Obesity**	Underweight	2.4	16.000 (0.791-323.701)	0.071	14.445 (0.673-310.198)	0.088	
		Normal	29.7	Ref		Ref		
		Overweight	44.3	2.057 (0.208-20.371)	0.537	1.938 (0.193-19.413)	0.574	
		Obesity	23.6	4.000 (0.399-40.059)	0.238	3.989 (0.395-40.241)	0.241	
**Macrosomia**	**Underweight**	Underweight	5.0	0	0.998	0	0.998	0.006
		Normal	53.7	Ref		Ref		
		Overweight	33.6	2.269 (0.714-7.209)	0.165	3.294 (0.765-14.187)	0.110	
		Obesity	7.7	2.827 (0.539-14.830)	0.219	1.501 (0.131-17.208)	0.744	
	**Normal**	Underweight	2.4	1.078 (0.388-2.996)	0.886	0.847 (0.2201-3.573)	0.821	
		Normal	50.4	Ref		Ref		
		Overweight	37.9	1.618 (1.190-2.199)	0.002*	1.677 (1.157-2.433)	0.006*	
		Obesity	9.3	1.639 (1.018-2.639)	0.042*	1.749 (1.002-3.053)	0.049*	
	**Overweight**	Underweight	1.3	2.65 (0.247-17.256)	0.503	0	0.999	
		Normal	41.7	Ref		Ref		
		Overweight	42.9	2.385 (1.321-4.306)	0.004*	2.054 (1.046-4.034)	0.037*	
		Obesity	14.1	2.209 (1.020-4.780)	0.044*	2.314 (1.001-5.348)	0.050*	
	**Obesity**	Underweight	2.4	0	0.999	0	0.999	
		Normal	29.7	Ref		Ref		
		Overweight	44.3	1.075 (0.330-3.507)	0.905	1.211 (0.331-4.436)	0.773	
		Obesity	23.6	3.185 (0.982-10.329)	0.054	2.714 (0.704-10.460)	0.147	

BMl, body mass index; SGA, small for gestational age; OR, odds ratio;95% CI, 95% confidence interval of the estimated trend.

a Univariate logistic regression compared with the referent (norma paternal BMI).

b Estimated using multivariate logistic regression. Model for SGA adjusted for maternal BMI, preeclampsia, maternal age. Model for macrosomia adjusted for maternal BMI, GDM, maternal weight gain.

c P values for Interaction showed the interaction effect of paternal obesity and maternal obesity on the risk of SGA/macrosomia.

*P < 0.05 was considered statistically significant.

### Development of a Nomogram for Predicting the Probability of Macrosomia and SGA

**Figure 3:**
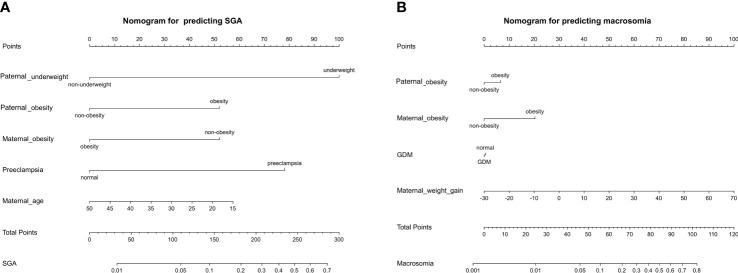
The nomogram was developed to predict the incidence rate of SGA **(A)** and macrosomia **(B)** based on the significant predictors in the multivariable analysis. Draw an upward vertical line from each variable axis to obtain the point of each variable. Calculate the sum of each variable score and draw an upward vertical line from the total score axis to obtain the predicted incidence of SGA or macrosomia.

The nomogram was developed using predictors from the multivariate analysis and significant variables pregravid and during pregnancy from univariate analysis to predict macrosomia and SGA risk. The nomogram for predicting SGA consisting of paternal underweight, paternal obesity, maternal obesity, preeclampsia, and maternal age (**Figure 3A**). The nomogram for predicting macrosomia consisting of paternal obesity, maternal obesity, GDM, maternal weight gain (**Figure 3B**). The total score calculated from the above variables was used to estimate status of SGA or macrosomia. These results suggested that the nomogram can be clinically useful in predicting the probability of the occurrence of macrosomia and SGA. This shows that paternal obesity is of great significance for the occurrence of both SGA and macrosomia.

## Discussion

The effects of maternal obesity on pregnancy and child health have been extensively studied, and systematic evaluations have found an increased incidence of pregnancy complications in pregnancy, including gestational diabetes (21), preeclampsia (22), hypertension, depression (23), cesarean section, preterm delivery, surgical site infections, and neonatal complications including perinatal death (24), macrosomia (25), and fetal defects. The impact of paternal obesity on pregnancy and child health has received less attention. Although most investigations have focused on the maternal environment, there is evidence that exposure to paternal obesity also predisposes offspring to metabolic disorders later in life (26). Obesity is a complex multifactorial disease involving genetic, epigenetic, and environmental factors (27, 28). The effect of epigenetic changes in sperm cells on fetal gene expression has been studied in animal models, and paternal environmental conditions have a negative impact not only on sperm but also on pregnancy success and offspring health. In reviewing the literature, some scholars (29) have emphasized the need to explore and recognize paternal contributions to the health of their offspring within the developmental origins of health and disease hypothesis and have referred to this new concept as the Paternal Origins of the Health and Disease paradigm (30) (POHaD). A better understanding of the preconception origins of disease through paternal exposure models will provide evidence-based public health recommendations for future fathers (30). However, so far, little is known about the potential role of paternal environmental impact on offspring’s health. Imprinted genes are well-known parents’ “conflict theory” that controls fetal growth. It is proposed that father-expressed genes (PEGs) promote nutrition from the mother, while mother-expressed genes (MEGs) restrict children’s use of resources (31). Imprinted genes are also thought to play an essential role in many biological processes and diseases, including intrauterine growth restriction (IUGR), obesity, and diabetes mellitus. Observational studies have found that increased paternal body mass index during pregnancy alters fetal cord blood methylation patterns, decreases neonatal IgM levels (32), is associated with delayed personal and social functioning in three-year-old children (33), increases the incidence of type II diabetes and insulin resistance (34), and is associated with the severity of obesity in childhood (35). Studies have found that obesity in the father during pregnancy alters birth weight (36–38) and increases the child’s susceptibility to metabolic syndrome (36), subfertility (39), fatty liver (40), kidney disease (41), and hypertension (42) while reducing the child’s cognitive function (43). It is widely believed that these factors have the potential to influence early embryonic and fetal growth and development, leading to the hypothesis that paternal obesity can influence pregnancy and child health outcomes long after pregnancy through epigenetic alterations. Paternal obesity has been shown to be associated with increased weight and body fat and metabolic disturbances in offspring prepubertal children (44). Recent studies have shown that paternal high-fat diet programs b-cell dysfunction in female rat offspring through epigenetic mechanisms (45). A similar paternal obesity effect was also found in the mouse model of paternal obesity. More and more evidence mainly come from animal experiments, which shows that Father also plays an essential biological role in fetal programming, but so far, only a few human epidemiological data support this concept. The clinical community still generally does not recognize the impact of father obesity on pregnancy and child health. This study will focus on the evidence of the effects of paternal obesity on pregnancy outcomes and fetal development to standardize and optimize paternal health to improve health outcomes in the next generation, creating a theoretical basis and enabling conditions for primary prevention of chronic disease.

### Effect of Paternal Obesity on Placental Development and Pregnancy Complications

The placenta is known to be a dynamic regulator of fetal nutrient transport and growth and is a unique organ because it is influenced by three genomes: the mother, the father, and the fetus. The paternal genome, especially the epigenome, plays a crucial role in the development and function of the placenta, which in turn regulates fetal growth (46). The interaction between paternal factors and placental development has been most extensively studied in preeclampsia. Recent data also suggest that paternal nutrition and weight status can alter placental function in offspring. Several research groups have reported that paternal BMI is the predictor of placental and birth weight in the offspring (13). Therefore, more studies are needed to fully understand the role of paternal nutritional status in shaping placental function. There is evidence that fathers play an essential role in the development of preeclampsia. The existence of a “paternal antigen” has been proposed and paternal obesity has also been suggested as a risk factor for preeclampsia (47). Fetal HLA-G variants from fathers increase immune incompatibility with mothers and are also significantly associated with preeclampsia in multiple pregnancies. Epidemiological, clinical, immunological, and genetic evidence supports the role of the father in the development of preeclampsia (47). The study demonstrates that paternal obesity in mice is associated with impaired embryonic development and significantly reduces fetal and placental weight (48). Placental and fetal growth retardation associated with paternal obesity is opposite to the effect of maternal obesity on pregnancy, which is significantly associated with the incidence of older than gestational age infants. Placental growth deceleration generally precedes fetal growth deceleration (49), and placental size has been identified as an independent determinant of intrauterine fetal growth and birth weight (50). Paternally expressed imprinted genes tend to enhance placental growth (51). Thus, imprinting errors or epigenetic changes during spermatogenesis may affect the placenta and subsequent fetal growth associated with paternal obesity. In mice’s high-fat diet-induced obesity model, paternal obesity was associated with reduced placental weight, fetal growth restriction, and altered placental expression and DNA methylation of genes related to lipid metabolism. In mice, hypermethylation of just one parentally expressed imprinted gene is sufficient to induce FGR (52). In addition, global methylation changes of non-imprinted genes and altered gene expression have also been found in FGR placentas (53). SGA (small for gestational age) is a major obstetric complication caused by placental dysplasia and is an important cause of perinatal mortality and morbidity. SGA is mainly due to placental insufficiency, which reduces the supply of nutrients and oxygen to the developing fetus (54). Fetal adaptation to this altered uterine environment results in permanent changes in glucose-insulin metabolism (55). Reduced hypothalamic satiety pathways in younger than fetal age children lead to programmed overeating and a self-perpetuating cycle of obesity (56). SGA infants are also associated with an increased risk of NCDs in offspring (57). Individuals with growth restriction are at increased risk of developing metabolic syndromes in later life, including obesity, hypertension, cardiovascular disease, and type 2 diabetes (58, 59). It has been shown that embryos from paternal obesity have a tendency for fertilization arrest, delayed preimplantation development, mitochondrial dysfunction, reduced blastocyst formation, reduced cell numbers, and abnormal cell lineage assignment to the trophoblastic ectoderm (TE) or inner cell mass (ICM) (60). Also, obese fathers have significantly smaller fetal development, delayed limb morphology, and a substantially smaller placenta (61, 62). In addition, paternal obesity increases the likelihood of metabolic syndrome in the offspring (63). Our study showed that paternal obesity was significantly associated with the incidence of preeclampsia, with a progressive increase in preeclampsia as paternal BMI increased in a linear association (P<0.001). In addition, paternal obesity is an independent predictor of both SGA (OR=2.866, 2.091-3.930) and macrosomia (OR=1.690, 1.168-2.444). All these results confirm the adverse effects of paternal obesity on placental development and pregnancy complications.

### Effect of Paternal Obesity on Fetal Development

At birth, both SGA and macrosomic infants are associated with increased fetal complications, including increased NICU and fetal mortality (64, 65). They also increase the risk of developing chronic diseases later in life. It should be noted that early signs of these chronic disease states have been shown in the offspring of animal models with obese fathers (66, 67). The study showed an increased likelihood of both SGA and macrosomia with paternal obesity (68), which may be why other studies have generally found no effect on average birth weight. The existing literature reports two diametrically opposite birth weight results for fetal development of obese fathers: macrosomia (69) and SGA (70), and both have statistical differences. McCowan et al. (70) found that obese men (BMI ≥ 30 kg/m^2^) were more likely to have SGA than non-obese men. Conversely, paternal underweight (<18.5 kg/m^2^) is independently associated with SGA (71).

In contrast, Yang et al. conducted a case-control study and found that overweight and obese fathers were significantly associated with the risk of macrosomia (72). After a study included linear interaction, it was found that paternal obesity weakened the positive correlation between maternal obesity and newborn birth weight. When the father is obese, the increase in average birth weight associated with maternal obesity is weakened (73). And those studies that assessed birth weight as a continuous measure did not find any consistent association, because the association between paternal obesity and SGA may be masked by the co-occurrence between paternal obesity and macrosomia (74). Chen et al. (75) showed that paternal BMI was significantly associated with birth weight, biparietal diameter, head circumference, abdominal diameter, abdominal circumference, and chest diameter of male offspring. Therefore, cohort studies are needed to confirm whether the findings are similar in humans and investigate the effect of paternal BMI on the distribution of birth weight beyond mean differences and bipolar categories. We use the ultrasound measurements to describe the body shape of newborns, because it is known that the birth process will affect some anthropometric parameters immediately after birth (76) (e.g. head circumference, etc.). Our study showed that there was a positive linear relationship between paternal BMI and BPD, FL, APAD, TAD, AC, placental weight, and placental area (p for trend<0.05), but in the group of paternal obesity, there was no difference in the above indicators compared to normal controls. These results confirmed the above conjecture that high paternal BMI increased the frequency of unhealthy extremes, including macrosomia and SGA, but had a relatively small impact on the average. Our study also showed by logistic regression analysis that paternal obesity was the independent risk factor for the development of both SGA and macrosomia, which also confirmed the above theory. The interaction test showed that the effect of paternal obesity on SGA and macrosomia was significantly affected by maternal obesity. For maternal BMI normal and overweight, paternal obesity can significantly increase the occurrence of SGA and macrosomia. However, for maternal obesity, paternal BMI had little effect on the incidence of both SGA and macrosomia.

### Future Prospect

In many media and government campaigns, women remain the primary target for improving the health of their pregnancies and offspring (77) and ignore the contribution of the fathers. In reviewing available guidelines and recommendations, no country has yet issued separate specific guidelines for men (78). The lack of research interest in the role of fathers is evident in human studies, as most of the literature related to early exposure does not have a section on paternal influence. Epigenetic modifications are a continuous process, and some changes may be reversible. Experiments have shown that preconception dietary and exercise interventions improve sperm function and embryonic development in a paternally obese mouse model (79). Human studies have also shown that exercise can improve male fertility (80). Weight loss following bariatric surgery has also been shown to reshape sperm DNA methylation patterns (81). These findings provide new insights into the shared responsibility of parents for the intergenerational origins of obesity. There is an urgent need to define this new concept and its mechanistic basis in embryonic programming, identify effective interventions for obese parents before pregnancy, and optimize paternal health to improve the health outcomes of the next generation, creating exciting new opportunities for chronic disease prevention (82). Future comprehensive studies should include paternal epidemiology and epigenetic studies to understand the underlying intergenerational mechanisms of early human exposure. The paternal obesity status dramatically alters the sperm epigenome, which may have important implications for the susceptibility of offspring to metabolic diseases. In addition to clarifying the epigenetic mechanism of paternal obesity affecting intrauterine development, future research directions can also focus on whether pre-pregnancy diet and exercise intervention can improve and reverse the adverse effects on offspring, optimize the status of paternal obesity to improve the health outcomes of the next generation, and create opportunities for the prevention of chronic diseases.

### Strengths and Limitations

Our study has several advantages, such as a relatively large sample size, and our data cover the measurements of fetal growth, neonatal weight, neonatal body length, as well as placental area and weight entirely. In addition to the stratification of maternal BMI, we also studied the interactive effects of paternal obesity and maternal obesity on the incidence of SGA and macrosomia, which has not been seen in the previous studies. All participants were Chinese, minimizing genetic susceptibility differences in birth weight and gestational age. The advantage of our study also lies in its prospective and rigorous method. In analyzing the impact of paternal BMI on fetal development and pregnancy complications, we have controlled for parental age, maternal BMI, weight gain during pregnancy, and other influencing factors. Importantly, our study enables us to address the potential contribution of paternal obesity to a range of fetal anthropometric indicators and placental function, which is rare so far. Information on birth outcomes and maternal complications during pregnancy comes from medical records, minimizing misclassification. In this study, the maternal BMI was measured by instruments, but the paternal BMI was calculated by self-report. This is also a limitation that may increase the risk of bias and the risk of deviation. However, a previous study showed that the self-reported anthropometric index was accurate enough among European adults (83). One study showed that the average weight of young men changes by 0.6 kg over the course of a year (84), which is considered unlikely to change within a few months of obtaining paternal measurements.

## Conclusion

Our study showed that there was a positive linear relationship between paternal BMI and fetal BPD, FL, APAD, TAD, AC, placental weight, and placental area (trend P < 0.05), but there was no difference between obese father group and normal control group. These results confirm our conjecture that the high BMI of fathers increases the frequency of unhealthy extremes, including macrosomia and SGA, but has a relatively small impact on the average fetal birth weight. In addition, paternal obesity was significantly associated with the incidence of preeclampsia, macrosomia, and SGA. Although the effect of paternal obesity on SGA and macrosomia was significantly affected by maternal obesity, paternal obesity was still an independent predictor of SGA and macrosomia. All these results confirm the adverse effects of paternal obesity on placental development and pregnancy complications. Optimizing the paternal BMI will help improve the health of the next generation.

## Data Availability Statement

The original contributions presented in the study are included in the article/**Supplementary Material**. Further inquiries can be directed to the corresponding author.

## Ethics Statement

The studies involving human participants were reviewed and approved by The Ethics Committee of the International Peace Maternity and Child Health Hospital (reference number GKLW 2021-23). The patients/participants provided their written informed consent to participate in this study.

## Author Contributions

HH contributed to the design of the study, collection, interpretation of data, and revising the manuscript. JL participated in the design of the study, analyzed the data and drafting the manuscript. WG was responsible for the collection and interpretation of data. WG and JL conceived the study and reviewed/edited the manuscript. All authors contributed to the article and approved the submitted version.

## Funding

This study was funded by National Key Research and Development Program of China (Grant Number: 2019YFA0802604) and National Natural Science Foundation of China (Grant Number: 81861128021). The funders had no role in the study design, data collection, data analysis, data interpretation, or writing of the report. The corresponding author had full access to all data in the study and made the final decision to submit the study for publication.

## Conflict of Interest

The authors declare that the research was conducted in the absence of any commercial or financial relationships that could be construed as a potential conflict of interest.

## Publisher’s Note

All claims expressed in this article are solely those of the authors and do not necessarily represent those of their affiliated organizations, or those of the publisher, the editors and the reviewers. Any product that may be evaluated in this article, or claim that may be made by its manufacturer, is not guaranteed or endorsed by the publisher.
